# Ethnicity and neighbourhood deprivation determines the response rate in sexual dysfunction surveys

**DOI:** 10.1186/s13104-015-1387-2

**Published:** 2015-09-04

**Authors:** Lasantha S. Malavige, Pabasi Wijesekara, Dhanesha Seneviratne Epa, Priyanga Ranasinghe, Jonathan C. Levy

**Affiliations:** Oxford Centre for Diabetes, Endocrinology and Metabolism, Nuffield Department of Clinical Medicine, University of Oxford, Oxford, OX3 7LJ UK; Genetech Research Institute, Colombo, Sri Lanka; Ministry of Health Care and Nutrition, Colombo, Sri Lanka; Department of Pharmacology, Faculty of Medicine, University of Colombo, Colombo, Sri Lanka

**Keywords:** Sexual dysfunction, Diabetes mellitus, South Asians, Europids, Response rate

## Abstract

**Background:**

Self-administered questionnaires provide a better alternative to disclose sensitive information in sexual health research. We describe the factors that determine the positive response (initial recruitment) to an initial invitation and subsequent completion of study to a postal questionnaire on sexual dysfunction.

**Methods:**

South Asians (SA) and Europids with and without diabetes (DM) were recruited from GP clinics in UK. Men who returned the properly filled consent form (‘recruited-group’) were sent the questionnaire and those who returned it were considered as the ‘completed-group’. Index of Multiple Deprivation Scores (IMDs) were generated using UK postcodes. We calculated the recruitment rate and completion rate of the recruited and the study-completed groups respectively.

**Results:**

Total approached sample was 9100 [DM: 2914 (32 %), SA: 4563 (50.1 %)]. Recruitment rate was 8.8 % and was higher in Europids and in patients with DM. Mean IMDs for the recruited group was 20.9 ± 11.9, and it was higher among recruited SA compared to Europids (p < 0.001). Mean IMDs was higher in the recruited group compared to non-recruited (p < 0.01). All four recruited groups (SA/Europid and DM/non-DM) had lower IMDs compared to non-recruited. Completion rate was 71.5 % (n 544) (SA: 62.3 %, Europids: 77.4 %; p < 0.05).

**Conclusion:**

Recruitment for postal sexual health surveys is positively influenced by presence of investigated disease, older age, being from lesser deprived areas and Europid ethnicity. Furthermore, Europids were more likely to complete survey than South Asians irrespective of disease status.

## Background

Self administered questionnaires provide a better alternative to disclose sensitive information for study participants. Amongst the different self-administrative survey methods, postal questionnaires have long been used successfully to evaluate sexual dysfunction and sexual behaviour. In comparison to face-to-face and telephone interviews, the higher levels of privacy and confidentiality offered in postal methods, provides the participant a better opportunity to reveal truthful information [1–3]. Computer assisted self-interviews are an emerging new method of survey, however its superiority in response when compared to postal surveys remain inconclusive [1, 4].

Low response rates and the accompanying non-responder bias is a common problem in postal surveys, affecting the generalisabilty and validity of the findings [5]. It is important therefore, to identify how responders differ from non-responders. Questionnaire topic, length, sensitivity, pre-notification, incentives and intense follow up are well known determinants of response rate [6]. Responder related factors such as age, intelligence, social class and level of education are also known to influence the response rate [7–9]. At present there is only limited data available on the factors affecting the response rate in postal surveys on sexual dysfunction and sexual behaviour. The GSSAB study (Global Study of Sexual Attitudes and Behaviours) demonstrated that there is a possible socio-cultural inhibition influencing response amongst sexually conservative groups [10]. However, to our knowledge, a comparison of response rates between the South Asian and Europid ethnic groups has not been described previously in the sexual health epidemiology.

The Oxford Sexual Dysfunction Study (OSDS) was a multi centred GP practice based study that describes sexual dysfunction in men with and without diabetes of South Asian and Europid ethnic origin living in the UK. One of the primary objectives of this study was to evaluate the feasibility of using validated postal questionnaires to assess sexual dysfunctions and their clinical, socioeconomic and lifestyle associations. The present report aims to describe the factors that determine the positive response (initial recruitment) to an initial invitation and subsequent completion of study to a postal questionnaire on sexual dysfunction.

## Methods

### Study population and sampling

Thirty-seven (GP) clinics from eight primary care trusts (PCTs) in the UK were invited for the OSDS. The OSDS is a large survey which aimed to evaluate the prevalence and associations of sexual dysfunction among males of South Asian and Europid origin, both with and without diabetes resident in the UK [11]. Ethical approval for the study was obtained from the Oxfordshire Research Ethics Committee C. Research Governance approval for the study was granted by the regional PCTs of Ealing, Brent, Luton, Redding, Slough, Swindon and Coventry. According to recent national population data, South Asians represent 4–5 % of the UK population [12]. Hence a selective approach was used to select GP practices in order to recruit a larger population of South Asians. Geographical areas with a considerably high South Asian population were identified. In these areas, the GP practices with higher numbers of registered South Asians were selected and invited to participate in the study. The selective approach was guided by local collaborators in the regional hospitals. The study included patients with and without diabetes of South Asian and Europid origin.

Patients with diabetes were selected as follows. In the GP practices that agreed for collaboration, we selected all male patients in the practice’s diabetes registry between 21 and 70 years, and stratified this sample into five age categories (21–30, 31–40, 41–50, 51–60 and 61–70). Men with diabetes in each age category were sub-categorized into Europid and South Asian ethnicities. When the ethnicity and first language were not recorded in the practice data base, the researcher allocated the most likely ethnicity and the first language for the particular case, based on the name/surname and recommendation of the practice doctor. The accuracy of the allocated ethnicity was verified later by comparing with the participant reported ethnicity in the returned questionnaire. The same method was also applied in patients without diabetes.

Patients without diabetes were selected as follows. Diabetes is approximately 3–5 times more common among South Asians [13, 14]. Therefore, we hypothesised that the proportion of South Asians in patients without diabetes would be smaller than in those with diabetes. Further, we assumed that the response rate would be lower among those without diabetes compared to the patients with diabetes, based on previous similar studies in patients with respiratory diseases/symptoms [15]. For these reasons, we approached twice as many males without diabetes in the relevant age categories above as the control group (as shown below). The number of South Asian and Europid men in the sample of patients with diabetes were considered X and Y respectively. A four times bigger random sample [4(X + Y)] of men without diabetes was drawn from the GP database in each age category as the Temporary Selection Sample (to ensure adequate representation of South Asians). This temporary selection sample was divided into South Asians without diabetes and Europids without diabetes lists using a similar procedure as for the patients with diabetes. From each of these lists of those without diabetes sorted in alphabetical order a secondary random sample of 50 % was obtained, ensuring the final invited control group sample size was twice that of the disease group. These samples together (males with and without diabetes) were termed as the Initial Approach Sample. The exclusion criteria included men who had spinal cord damage, undergone surgery of the prostate and/or pelvic irradiation and diagnosed with serious psychiatric conditions.

We invited the selected men with and without diabetes, with endorsement letters from the GP, to participate in the study, as studies have shown that this increases participation [16]. The invitation pack contained a personalised invitation letter, information sheet and the consent form. These documents were originally developed in English language and translated into five South Asian languages; Hindi, Punjabi, Sinhalese, Tamil and Urdu using translation–retranslation technique [17]. Those who did not respond the invitation within a fortnight were sent a 2nd invitation. This second invitation was sent out only to a randomly selected sub-group (diabetic group: 36 %, control group: 30 %) of those of did not respond to 1st invitation. The men who returned the properly filled consent form with the consent to participate were recruited for the study and others were included in the non recruited group. The recruited group received the two booklet questionnaire designed for the study. The questionnaire was also available in the above mentioned five South Asian languages, upon request by the participant on the consent form [17]. The non-responding recruited subjects received a first and a second reminder, each within 2 week intervals. The group who returned the completed questionnaire was considered the “completed group”.

### Data collection and analysis

The Index of Multiple Deprivation scores (IMD score) of all men were generated using the post codes, through the Economic and Social Research Council (ESRC) census programme, UK (www.census.ac.uk). The IMD brings together 37 different indicators of deprivation including; income, employment, health and disability, education, skills and training, barriers to housing and services, living environment and crime. The higher IMD scores indicate lesser deprivation compared to the areas with lower scores.

We calculated the response rates for the recruited and the study completed group as below. These rates were compared between South Asian and Europid, men with and without diabetes using Chi square analysis.

$${\text{Recruitment Rate }} = \frac{\text{Total number of men who returned the completed consent form}}{{{\text{Total number of }}\left( {{\text{approached men }}{-}{\text{ undelivered mail}}} \right)}}$$

$${\text{Completion Rate }} = \frac{\text{Total number of men who returned the completed questionnaire}}{\text{Total number of men recruited for the study}}$$

Data was analysed using the SPSS version 17 (Chicago. IL, USA). Mean values of age and IMD scores for the four groups (men with and without diabetes; South Asian and Europid) within the approached, recruited and study completed groups were compared using analysis of variance (ANOVA). Mean age and IMD score for the recruited group was compared with the non recruited group and completed group was compared with the non completed group within each of the above four groups as well using ANOVA.

A binary logistic regression analysis was performed ‘successful recruitment’ as the dichotomous dependent variable (0 = not recruited; 1 = recruited) and using age (continuous), IMD score (continuous), presence of diabetes (binary, 0 = no; 1 = yes) and ethnicity (binary, 0 = South Asian; 1 = Europid) as the independent variables. A similar binary logistic regression analysis with above independent variables was also performed separately for study completion using ‘study completion’ as the dichotomous dependent variable (0 = not completed; 1 = completed).

## Results

### Sample characteristics

**Fig. 1:**
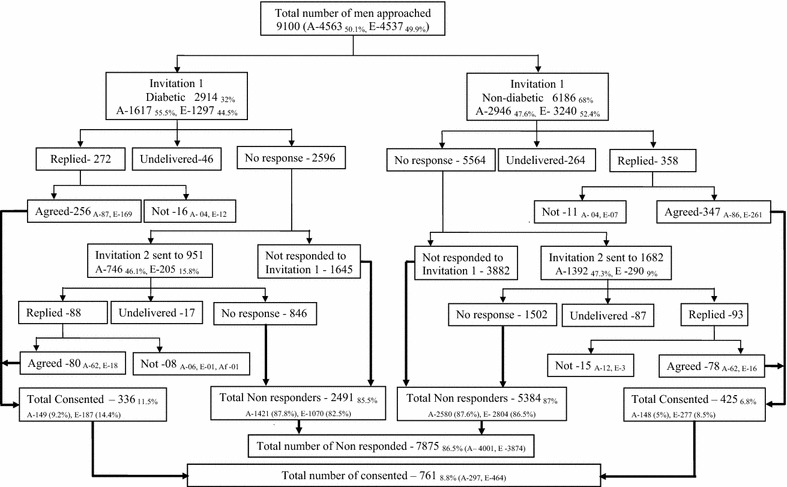
Summarized recruitment process (*A* South Asians, *E* Europids, *Af* Afro-Caribbean)

Twenty-five GP practices of the 37 GP practices invited from the 8 PCTs agreed to take part in the study. There were differences in the recruitment and completion rates between the practices (Table 1). The total approached sample was 9100 with 2914 (32 %) patients with diabetes mellitus and 6186 (68 %) males without diabetes. According to allocated ethnicity, 4563 (50.1 %) men were South Asians and 4537 (49.9 %) were Europids (Fig. 1). The allocated ethnicity was 91.5 % accurate (498/544) when verified with the participants’ reported ethnicity in the questionnaires, with 34 participants reporting an ethnicity other than Europid or South Asian.

**Table 1:** Participant recruitment and completion rates of the questionnaire in the 25 GP practices

PCT	GP practice	Approached^a^ (%)	Diabetic		Non diabetic		Recruited^d^ (%)	Completed^d^ (%)
			Recruited^b^	Completed^c^	Recruited^b^	Completed^c^		
Brent	The Surgery Wembley Centre for Health	259 (2.8)	12	6	13	5	25 (9.6)	11 (44.0)
	Wembley Park Medical Centre Wembley	622 (6.8)	4	4	26	15	30 (4.8)	19 (63.3)
Coventry	Swanswell Medical Centre	111 (1.2)	2	1	3	3	5 (4.5)	4 (80.0)
	Central Medical Centre Coventry	317 (3.5)	12	9	17	10	29 (9.2)	19 (65.5)
	Paradise Medical Centre	169 (1.9)	4	3	0	0	4 (2.4)	3 (75.0)
Ealing	Jubilee Gardens Medical Centre Middlesex	495 (5.4)	13	8	15	9	28 (5.7)	17 (60.7)
	The Town Surgery Middlesex	357 (3.9)	12	6	5	2	17 (4.8)	8 (47.0)
	The Barnabas Medical Centre	389 (4.3)	17	15	20	19	37 (9.5)	34 (91.9)
	Hanwell Health Centre	30 (0.33)	3	2	0	0	3 (10.0)	2 (66.7)
	Belmont Medical Centre	388 (4.3)	19	13	20	12	39 (10.1)	25 (64.1)
	Ealing Park Health Centre	286 (3.1)	6	5	23	18	29 (10.1)	23 (79.3)
Luton	Blenheim Medial Centre	600 (6.6)	18	13	24	18	42 (7.0)	31 (73.8)
	The Link Surgery Luton	134 (1.5)	6	5	4	3	10 (7.5)	8 (80.0)
Oxfordshire	Donnington Health Centre	143 (1.6)	1	1	8	6	9 (6.3)	7 (77.8)
	White House Surgery	198 (2.2)	10	7	20	17	30 (15.2)	24 (80.0)
Redding	The Loddon Vale Practice	410 (4.5)	28	18	18	16	46 (11.2)	34 (73.9)
	Grovelands Medical Centre	441 (4.8)	18	11	30	24	48 (10.9)	35 (72.9)
	Wexham Road Surgery	571 (6.3)	15	8	31	20	46 (8.1)	28 (60.9)
	The Riverside Surgery	545 (6.0)	20	17	32	28	52 (9.5)	45 (86.5)
Slough	Chapel Medical Practice	235 (2.6)	5	3	11	7	16 (6.8)	10 (62.5)
	The Avenue Medical Centre	463 (5.1)	22	19	26	20	48 (10.4)	39 (81.2)
	Kumar Medical Centre	510 (5.6)	21	10	22	10	43 (8.4)	20 (46.5)
	Manor Park Medical Centre	724 (8.0)	21	17	21	14	42 (5.8)	31 (73.8)
	Crosby House Surgery	411 (4.5)	19	14	8	6	27 (6.6)	20 (74.1)
Swindon	Eldene Surgery	392 (4.3)	28	24	28	23	56 (14.3)	47 (83.9)
	Total	9100 (100.0)	336	239	425	305	761 (8.8)^e^	544 (71.5)

^a^Percentages for total number of men approached in each GP practice is calculated from the total number of men approached for the study

^b^Recruited: number of men consented to take part in the study

^c^Completed: number of recruited men who returned the completed questionnaire

^d^Percentages for total recruited and completed men are calculated from the total number of men approached from each GP practice

^e^After adjusting for undelivered mail (n 414)

### Study recruited group

**Fig. 2:**
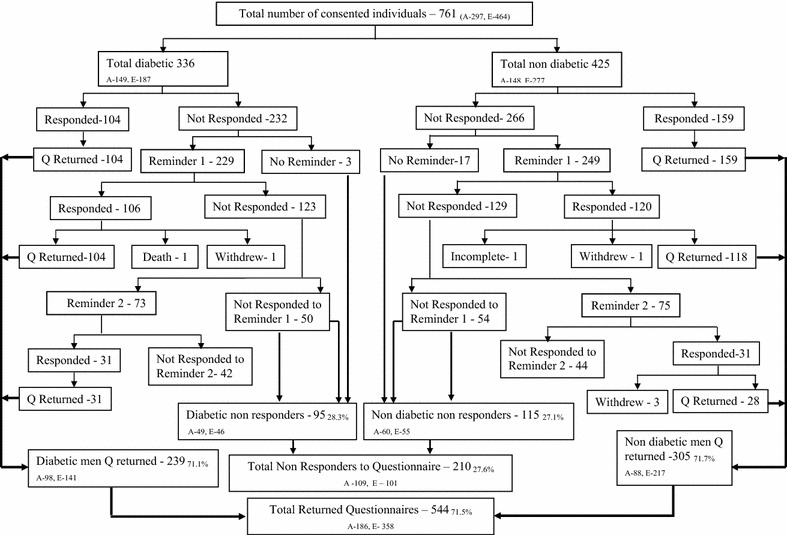
Summarized study completion process (*A* South Asians, *E* Europids, *Q* Questionnaire)

Seven hundred and sixty one men consented and were recruited for the study (Fig. 1). The overall recruitment rate for the study was 8.8 % after adjusting for undelivered mail (n 414). Recruitment rate for the first invitation was 6.9 % and it was significantly higher among Europids (9.9 %) compared to South Asians (4 %) and among patients with diabetes (9 %) compared to those without (5.9 %). Recruitment rate for the second invitation was 6.2 % and it was 6 and 7.2 % for the South Asian and Europid ethnic groups respectively (p = 0.67). The recruitment rate for second invitation in the males with diabetes (8.6 %) was significantly higher compared to those without diabetes (4.9 %) (p = 0.001). Recruitment rate was significantly higher among Europids in comparison to South Asians irrespective of their diabetes status (p < 0.001) (Table 2). Similarly, the rate was significantly higher among patients with diabetes compared to those without diabetes irrespective of the ethnicity (Table 2). Figures 1 and 2 summarise the process of recruitment, follow up and completion of the study.

**Table 2:** Mean age and IMD scores for the Approached, Recruited and Study completed men

	All	Men with Diabetes			p*	Men without Diabetes			p*
		Total	South Asians	Europids		Total	South Asians	Europids	
Approached group									
Number (%)	8686	2851 (32.8)	1580 (36.5)	1271 (29.1)		5835 (67.2)	2744 (63.5)	3091 (70.9)	
Mean age ± SD	53.5 ± 11.1	55.5 ± 10.4	54.2 ± 10.4	56.6 ± 10.3	<0.001	52.6 ± 11.2	50.9 ± 11.3	53.6 ± 11.1	<0.001
IMD score ± SD	22.3 ± 11.9	21.9 ± 12.4	25.8 ± 11.6	19.1 ± 12.3	<0.001	22.4 ± 11.7	26.4 ± 11.5	20.1 ± 11.2	<0.001
Recruited group									
Number (%)	761 (8.8)	336 (11.8)	149 (9.4)	187 (14.7)	<0.001	425 (7.3)	148 (5.4)	277 (9.0)	<0.001
Mean age ± SD	56.8 ± 9.3	58.0 ± 9.2	58 ± 9.7	58 ± 8.9	0.99	55.9 ± 9.3	56 ± 9.9	55.8 ± 9.1	0.83
IMD score ± SD	20.9 ± 11.9	21.5 ± 12.4	25.3 ± 10.9	18.7 ± 12.6	<0.001	19.8 ± 11.5	24.8 ± 11	17.5 ± 10.9	<0.001
Study completed group									
Number (%)	544 (71.5)	239 (71.1)	98 (65.8)	141 (75.4)	0.47	305 (71.8)	87 (58.5)	218 (78.7)	<0.001
Mean age ± SD	57.1 ± 9.3	58 ± 9.2	59.2 ± 9.3	57.8 ± 9.3	0.25	55.9 ± 9.3	57.2 ± 9.9	56 ± 8.9	0.32
IMD score ± SD	19.9 ± 11.9	21.5 ± 12.4	25.4 ± 11.6	18.5 ± 12.3	<0.001	19.8 ± 11.5	23.5 ± 10.3	17.4 ± 11.2	<0.001

*SD* standard deviation

* Comparison of South Asian and Europid ethnicities within the diabetic and non diabetic groups

Mean age of the recruited men was 56.8 (±9.3) years. The mean age difference between the recruited males with diabetes (58 years) and males without diabetes (55.9 years) was 2.1 years (p < 0.01) (Table 2). Mean IMD score for the recruited group was 20.9 (±11.9). Mean IMD was significantly higher among recruited South Asians compared to Europids (p < 0.001) (Table 2). The difference of the mean IMD score between the recruited males with and without diabetes was not statistically significant (p = 0.053). Compared to the non recruited group, the recruited group was significantly older (56.9 ± 9.3 vs 53.1 ± 11.2) (p < 0.001). Mean IMD score difference between the recruited and non-recruited groups were significant (20.9 ± 11.9 vs 22.5 ± 11.9) (p < 0.01). All four groups (South Asian/Europid and with/without diabetes) of recruited men had lower IMD scores indicating lesser deprivation compared to non-recruited. However, this result was statistically significant only among males without diabetes of Europid ethnic origin (Table 3).

**Table 3:** Comparison of recruited/non-recruited and study completed/non-completed groups

	Mean ± SD		p*	Mean ± SD		p**
	Recruited	Non-recruited		Completed	Non- completed	
Diabetic						
South Asians						
Age	58 ± 9.7	53.6 ± 10.3	<0.001	59.2 ± 9.3	56.1 ± 10.2	0.07
IMD score	25.3 ± 10.9	25.9 ± 11.7	0.53	25.4 ± 11.6	25.3 ± 9.9	0.96
Europids						
Age	58 ± 8.9	56.3 ± 10.5	<0.05	57.8 ± 9.3	58.6 ± 7.5	0.56
IMD score	18.7 ± 12.6	19.2 ± 12.2	0.62	18.5 ± 12.3	19.5 ± 13.6	0.66
Non-diabetic						
South Asians						
Age	56 ± 9.9	50.6 ± 11.3	<0.001	57.2 ± 9.9	54.3 ± 9.7	0.08
IMD score	24.8 ± 11.0	26.5 ± 11.6	0.07	23.5 ± 10.3	26.7 ± 11.9	0.09
Europids						
Age	55.8 ± 9.1	53.4 ± 11.2	<0.01	56 ± 8.9	55.3 ± 9.8	0.62
IMD score	17.5 ± 11.0	20.4 ± 11.2	<0.001	17.4 ± 11.2	17.9 ± 10	0.74

* Comparison of recruited vs non recruited men

** Comparison of study completed vs not completed men

### Study completed group

Of the 761 (recruited group) who were sent the questionnaire, 263 returned the properly filled questionnaire upon receiving the two booklets and 498 did not return the questionnaire (Fig. 2). The completion rate for the study after two reminders was 71.5 % with 544 men completing the study. Overall 62.3 % South Asians and 77.4 % Europids completed the study. The completion rate was significantly higher among the Europids (p < 0.001). The proportion of males without diabetes completing the study was higher (71.8 %) than the males with diabetes (71.1 %) (p 0.66). Table 2 describes the demographic data for the approached, recruited and study completed groups. Mean age of the approached sample was 53.5 ± 11.1 years, with a mean age difference between the patients with and without diabetes of 2.9 years (P < 0.001). South Asian men in both with and without diabetes groups of the approached sample were younger compared to Europids (Table 2). Mean IMD score of the approached sample was 22.3 ± 11.9, while the IMD scores for those with and without diabetes were 21.9 ± 12.4 and 22.4 ± 11.7 respectively (p = 0.07). Europids had a significantly lower mean IMD score in the approached, recruited and study completed groups compared to the South Asians (p < 0.001) indicating lesser deprivation (Table 2).

Mean age of the study completed group was 57.1 ± 9.3 years. Mean age difference between the men with and without diabetes was 2.1 years (p < 0.01). We observed a trend for the study completed South Asians to be older than the Europids within both groups of males with and without diabetes (Table 2). Mean IMD score for the study completed groups was 19.9 ± 11.9 and was even lesser than that for the recruited group suggesting the likelihood for more affluent group of people to complete a postal questionnaire study (Table 2). Males with and without diabetes reported 21.5 ± 12.4 and 19.8 ± 11.5 as their mean IMD scores respectively (p = 0.053). The difference of this score between the two ethnic groups was statistically significant (p < 0.001) indicating the Europid ethnic group to be less deprived than the South Asian group (Table 2). The study completed men were older (p = 0.65) and the mean IMD scores were lower (p = 0.55) compared to the men who did not complete the study (Table 3).

### Logistic regression analysis

In the logistic regression analysis on study recruitment/study completion, the overall models were statistically significant and the Cox & Snell R-Square and Nagelkerke R Square values were 0.021/0.04 and 0.045/0.057 respectively. The results indicate that presence of investigated disease (OR 1.74), older age (OR 1.03) and being from lesser deprived areas and Europid ethnicity (OR 1.15), all significantly increased the likelihood of study recruitment (Table 4). However, study completion was influence only by Europid ethnicity (OR 1.96) (Table 4).

**Table 4:** Binary logistic regression analysis on study recruitment and study completion

Co-variants	Odds ratio (95 % CI)	
	Study recruitment	Study completion
Age	1.03 (1.02–1.04)*	1.02 (0.99–1.04)
IMD score	0.98 (0.97–0.99)*	0.98 (0.9–1.00)
Presence of diabetes	1.75 (1.45–2.08)*	0.82 (0.56–1.21)
Europid ethnicity	1.15 (1.05–1.25)*	1.96 (1.29–2.96)*

* p < 0.001

## Discussion

The difference between the responders and non responders with regard to the four factors we aimed at investigating was most apparent during the recruitment phase. The recruited group was significantly older and were from lesser deprived areas compared to the non recruited group. Europids and the patients with diabetes were more likely to consent for a postal survey. After consenting to participate in the study, completion appeared to be independent of age, area based deprivation and presence of diabetes. However, Europids were more likely to complete the survey than South Asians in both groups of males with and without diabetes.

Ethnicity has been recognised as a determining factor in most health related issues, including sexual health [18, 19]. Although research is limited comparing the response rates between the South Asian and Europid ethnic groups to a mail survey, studies have shown that ethnic minorities are significantly less likely to respond a mailed questionnaire than a telephone survey [20]. Linguistic difficulties, socio cultural influences on decision making, feeling of not belonging to the British society and social class could have been the potential disincentives for South Asian participation [21]. Whilst these factors remain potential determinants of South Asian participation, not being approached by the researchers is another common reason for lack of participation, due to increased cost and time associated with their inclusion particularly in relation to language barrier [22]. However, in our study there was equality in the approach by ethnicity the language barrier was also addressed. Nevertheless, the literacy rate among the South Asian ethnic group in the UK is lower. Thus, the lower literacy rate could also have been a detrimental factor for the response rate reported among this ethnic group, even with the language barrier excluded. In addition answering a postal questionnaire also needs a reasonable level of ability to read, comprehend and write. Therefore, South Asian ethnicity appear to be a negative contributory factor for the response rate of a postal survey on sexual health at both recruitment and completion stages, compared to the Europids, in the UK.

Associations between socioeconomic inequalities and health in the UK have been described in the past. In a closely comparable study, the response rate for a mailed questionnaire about the views of the general population on NHS has been higher among the people who lived in the lesser deprived areas; as determined by the Jarmon score area based deprivation [23]. Studies done outside the UK also provides supportive evidence [24]. However, the evidence is limited in the literature to support any relationship between the socio-economic status and the response rate to a postal questionnaire on sexual dysfunction. The disparity observed between the area based socio-economic statuses of Europid and South Asian ethnicities in the approached sample was comparable with the individual based socio economic status of the general population in the UK. However, even though the South Asians reported higher socio economic deprivation, during data analysis of the study completed group we found the level of education to be higher among the South Asians (these data is not presented in this paper) compared to the Europids. In line with our findings, Bhopal et al. have reported the South Asians to be advantaged in university education compared to Europeans in the UK [25].

Willingness to consent for research is generally variable among the public. Among the people with medical diseases, willingness to consent for research is arguable. A study done outside the UK reported a sample of women seen by the health care practitioner for sexually transmitted diseases to have been significantly less likely to respond to a postal data collection on sexual history and sexual behaviour compared to women who were seen for contraceptive advice [26]. However, these findings are not comparable with our findings with regard to the disease involved. In contrast the respiratory health epidemiology suggests the possibility of people who suffered more of the disease/symptoms to respond to a respiratory questionnaire [15]. This supports our observation of a higher response rate amongst the patients with diabetes. Furthermore, in line with our findings, responders have been older in several studies done both in the UK and elsewhere [23, 27, 28]. Lack of time available with the younger population, increased rates of moving and the less awareness of social responsibility compared to the older population may have been contributory factors for this finding.

During the participant selection stage the name of the person was used as a guide to determine his ethnicity in the absence of ethnicity recording at the particular GP practice. Recording the self ascribed ethnicity and the first language of all patients in the GP registry is a new introduction to the NHS system in the UK, under clinical directed enhanced services (DES). However, we experienced under reporting of ethnicity and the first language in most of the practice databases. Considering the South Asian names to ascertain their ethnicity has long been in use and considered a reliable alternative [29, 30]. We report 91.5 % accuracy in determining the ethnicity by names for this study, although it was time consuming.

The main limitation of the study was the low-recruitment rate (8.8 %) observed. Surveys on sensitive subjects such as sexual health, usually reports a lesser response rate regardless of the mode of administration or other characteristics of the participants such as age, gender or ethnicity [6, 20, 26]. In addition to addressing a sensitive issue, 50 % of the approached population for the current study was South Asians; an ethnic group among which the willingness to discuss the sexual issues is considered even less due to cultural reasons. In addition, male gender on its own has been identified as a factor for non response [27, 28]. The recruitment rate of this study may have influenced negatively by the above factors. However, personally addressed hand signed invitation letter by the GP, follow up, providing a second copy of the questionnaire at follow up, university sponsorship/collaboration, personalised cover letters and assurance of confidentiality used in the present study have been recognised in the previous studies as methods to increase response rate for postal questionnaires [27, 31]. Furthermore, the GP practice characteristics may also determine the response rate. The recruitment rate between the 25 GP practices varied considerably, from 0.1 to 15.2 %, in addition the completion rate also varied widely (0.7–12.1 %). Hence it is possible that the recruitment and completion rates were affected by the characteristics of the practice.

## Conclusion

Our results demonstrate that recruitment for postal surveys on sexual health is positively influenced by presence of the investigated disease, older age, being from lesser deprived areas and Europid ethnicity. However after recruitment, completion of the study appeared to be independent of age, area based deprivation and presence of diabetes. However, Europids were more likely to complete the survey than South Asians in both groups of males with and without diabetes.
